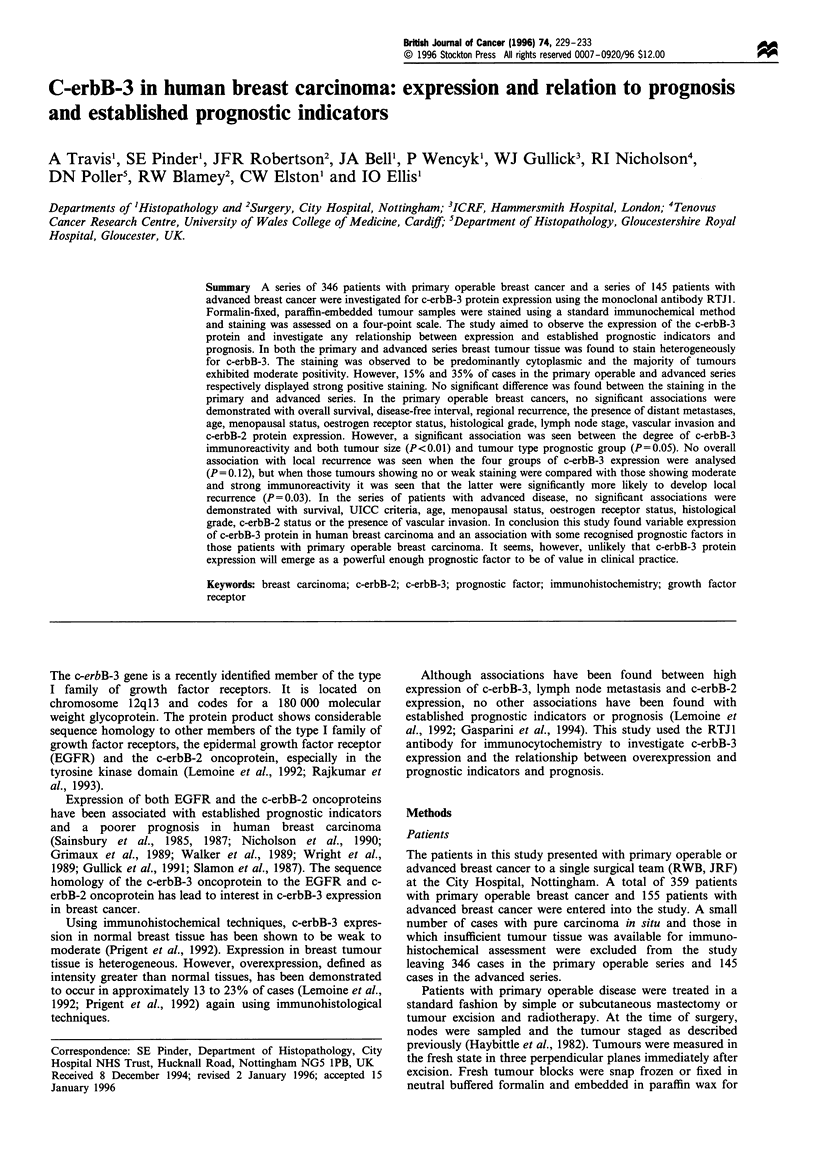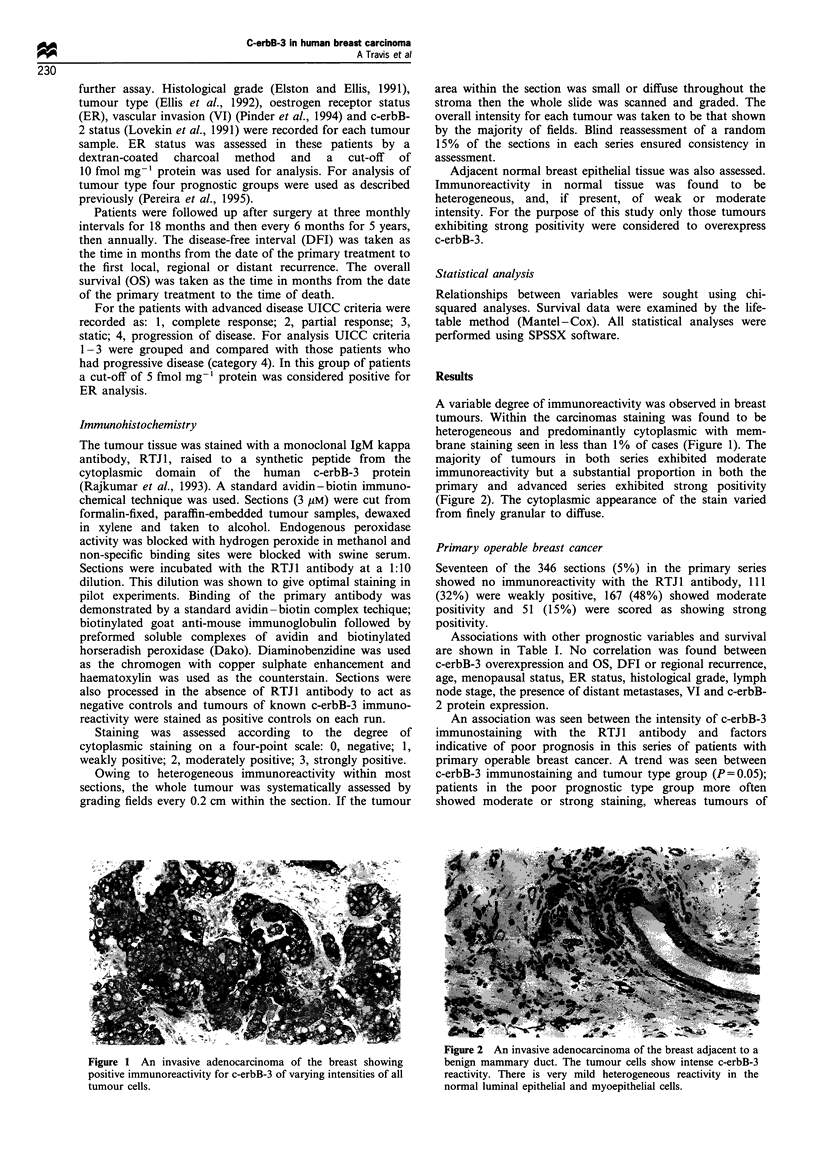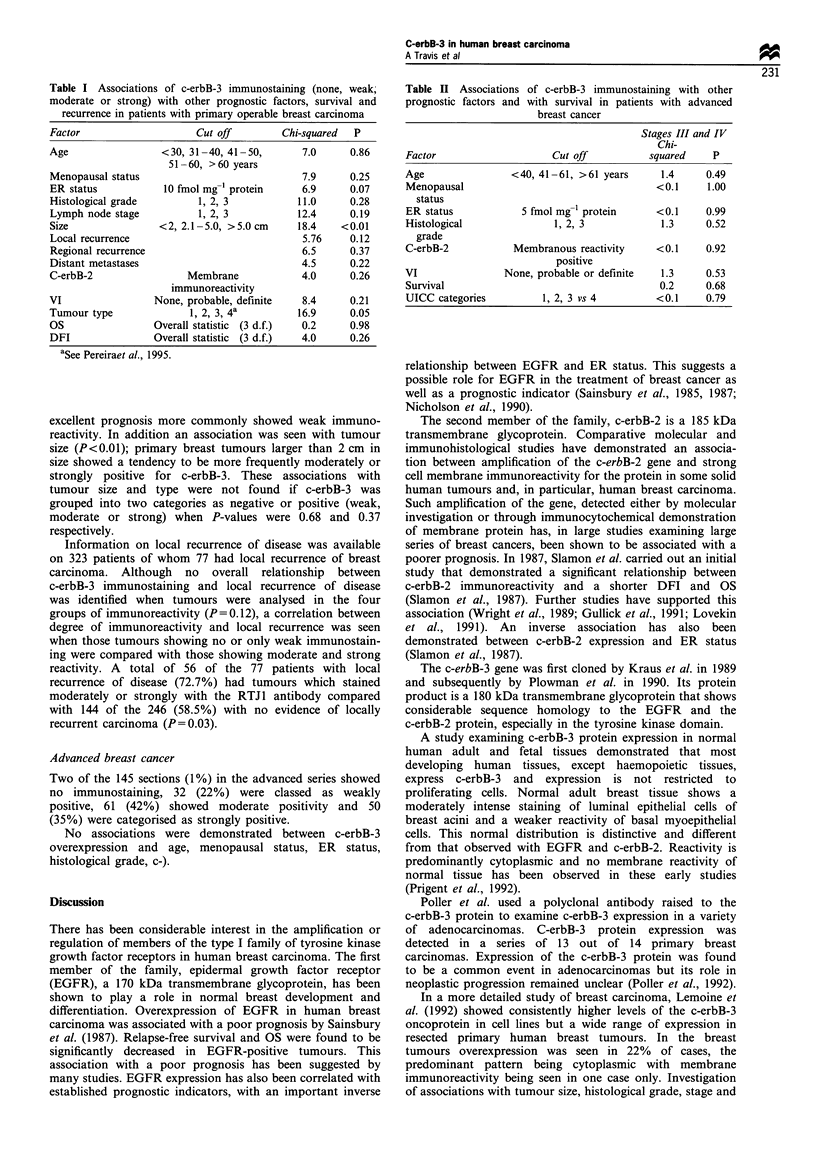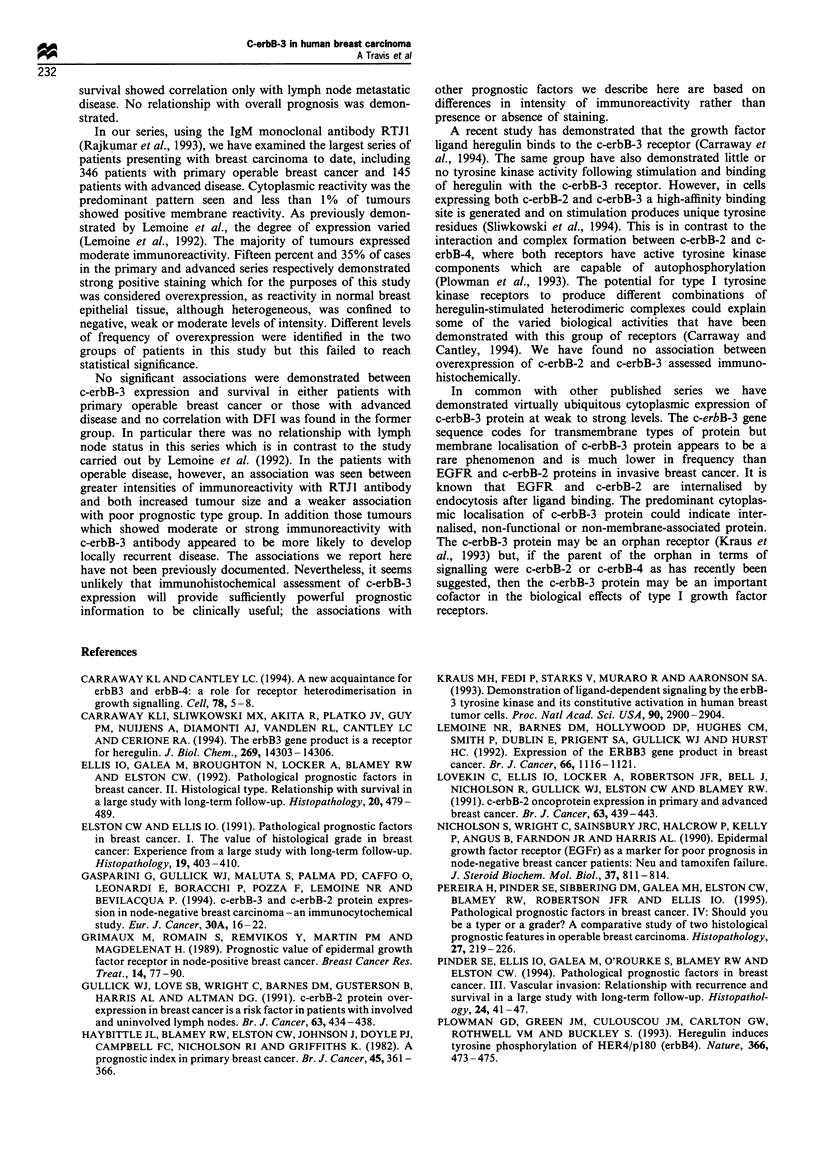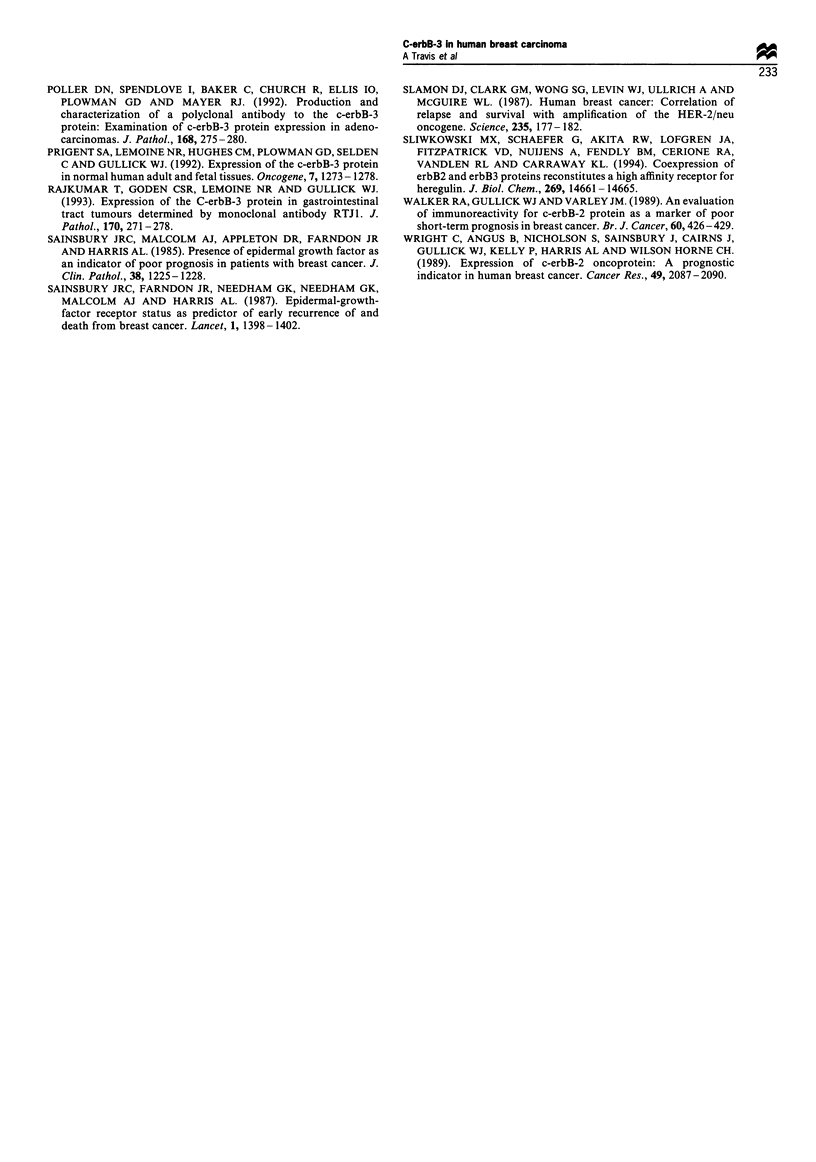# C-erbB-3 in human breast carcinoma: expression and relation to prognosis and established prognostic indicators.

**DOI:** 10.1038/bjc.1996.342

**Published:** 1996-07

**Authors:** A. Travis, S. E. Pinder, J. F. Robertson, J. A. Bell, P. Wencyk, W. J. Gullick, R. I. Nicholson, D. N. Poller, R. W. Blamey, C. W. Elston, I. O. Ellis

**Affiliations:** Department of Histopathology, City Hospital, Nottingham, UK.

## Abstract

**Images:**


British Journal of Cancer (1996) 74, 229-233
© 1996 Stockton Press All rights reserved 0007-0920/96 $12.00

### C-erbB-3 in human breast carcinoma: expression and relation to prognosis and established prognostic indicators

A Travis[1], SE Pinder[1], JFR Robertson[2], JA Bell[1], P Wencyk[1], WJ Gullick[3], RI Nicholson[4], DN Poller[5], RW Blamey[2], CW Elston[1] and IO Ellis[1]

*Departments of [1]Histopathology and [2]Surgery, City Hospital, Nottingham; [3]ICRF, Hammersmith Hospital, London; [4]Tenovus Cancer Research Centre, University of Wales College of Medicine, Cardiff; [5]Department of Histopathology, Gloucestershire Royal Hospital, Gloucester, UK.*

**Summary** A series of 346 patients with primary operable breast cancer and a series of 145 patients with advanced breast cancer were investigated for c-erbB-3 protein expression using the monoclonal antibody RTJ1. Formalin-fixed, paraffin-embedded tumour samples were stained using a standard immunochemical method and staining was assessed on a four-point scale. The study aimed to observe the expression of the c-erbB-3 protein and investigate any relationship between expression and established prognostic indicators and prognosis. In both the primary and advanced series breast tumour tissue was found to stain heterogeneously for c-erbB-3. The staining was observed to be predominantly cytoplasmic and the majority of tumours exhibited moderate positivity. However, 15% and 35% of cases in the primary operable and advanced series respectively displayed strong positive staining. No significant difference was found between the staining in the primary and advanced series. In the primary operable breast cancers, no significant associations were demonstrated with overall survival, disease-free interval, regional recurrence, the presence of distant metastases, age, menopausal status, oestrogen receptor status, histological grade, lymph node stage, vascular invasion and c-erbB-2 protein expression. However, a significant association was seen between the degree of c-erbB-3 immunoreactivity and both tumour size ($P<0.01$) and tumour type prognostic group ($P=0.05$). No overall association with local recurrence was seen when the four groups of c-erbB-3 expression were analysed ($P=0.12$), but when those tumours showing no or weak staining were compared with those showing moderate and strong immunoreactivity it was seen that the latter were significantly more likely to develop local recurrence ($P=0.03$). In the series of patients with advanced disease, no significant associations were demonstrated with survival, UICC criteria, age, menopausal status, oestrogen receptor status, histological grade, c-erbB-2 status or the presence of vascular invasion. In conclusion this study found variable expression of c-erbB-3 protein in human breast carcinoma and an association with some recognised prognostic factors in those patients with primary operable breast carcinoma. It seems, however, unlikely that c-erbB-3 protein expression will emerge as a powerful enough prognostic factor to be of value in clinical practice.

**Keywords:** breast carcinoma; c-erbB-2; c-erbB-3; prognostic factor; immunohistochemistry; growth factor receptor

The *c-erb*B-3 gene is a recently identified member of the type I family of growth factor receptors. It is located on chromosome 12q13 and codes for a 180 000 molecular weight glycoprotein. The protein product shows considerable sequence homology to other members of the type I family of growth factor receptors, the epidermal growth factor receptor (EGFR) and the c-erbB-2 oncoprotein, especially in the tyrosine kinase domain (Lemoine *et al.*, 1992; Rajkumar *et al.*, 1993).

Expression of both EGFR and the c-erbB-2 oncoproteins have been associated with established prognostic indicators and a poorer prognosis in human breast carcinoma (Sainsbury *et al.*, 1985, 1987; Nicholson *et al.*, 1990; Grimaux *et al.*, 1989; Walker *et al.*, 1989; Wright *et al.*, 1989; Gullick *et al.*, 1991; Slamon *et al.*, 1987). The sequence homology of the c-erbB-3 oncoprotein to the EGFR and c-erbB-2 oncoprotein has lead to interest in c-erbB-3 expression in breast cancer.

Using immunohistochemical techniques, c-erbB-3 expression in normal breast tissue has been shown to be weak to moderate (Prigent *et al.*, 1992). Expression in breast tumour tissue is heterogeneous. However, overexpression, defined as intensity greater than normal tissues, has been demonstrated to occur in approximately 13 to 23% of cases (Lemoine *et al.*, 1992; Prigent *et al.*, 1992) again using immunohistological techniques.

Correspondence: SE Pinder, Department of Histopathology, City Hospital NHS Trust, Hucknall Road, Nottingham NG5 1PB, UK
Received 8 December 1994; revised 2 January 1996; accepted 15 January 1996

Although associations have been found between high expression of c-erbB-3, lymph node metastasis and c-erbB-2 expression, no other associations have been found with established prognostic indicators or prognosis (Lemoine *et al.*, 1992; Gasparini *et al.*, 1994). This study used the RTJ1 antibody for immunocytochemistry to investigate c-erbB-3 expression and the relationship between overexpression and prognostic indicators and prognosis.

### Methods

### Patients

The patients in this study presented with primary operable or advanced breast cancer to a single surgical team (RWB, JRF) at the City Hospital, Nottingham. A total of 359 patients with primary operable breast cancer and 155 patients with advanced breast cancer were entered into the study. A small number of cases with pure carcinoma *in situ* and those in which insufficient tumour tissue was available for immunohistochemical assessment were excluded from the study leaving 346 cases in the primary operable series and 145 cases in the advanced series.

Patients with primary operable disease were treated in a standard fashion by simple or subcutaneous mastectomy or tumour excision and radiotherapy. At the time of surgery, nodes were sampled and the tumour staged as described previously (Haybittle *et al.*, 1982). Tumours were measured in the fresh state in three perpendicular planes immediately after excision. Fresh tumour blocks were snap frozen or fixed in neutral buffered formalin and embedded in paraffin wax for further assay. Histological grade (Elston and Ellis, 1991), tumour type (Ellis et al., 1992), oestrogen receptor status (ER), vascular invasion (VI) (Pinder et al., 1994) and c-erbB-2 status (Lovekin et al., 1991) were recorded for each tumour sample. ER status was assessed in these patients by a dextran-coated charcoal method and a cut-off of 10 fmol $mg^{-1}$ protein was used for analysis. For analysis of tumour type four prognostic groups were used as described previously (Pereira et al., 1995).

Patients were followed up after surgery at three monthly intervals for 18 months and then every 6 months for 5 years, then annually. The disease-free interval (DFI) was taken as the time in months from the date of the primary treatment to the first local, regional or distant recurrence. The overall survival (OS) was taken as the time in months from the date of the primary treatment to the time of death.

For the patients with advanced disease UICC criteria were recorded as: 1, complete response; 2, partial response; 3, static; 4, progression of disease. For analysis UICC criteria 1–3 were grouped and compared with those patients who had progressive disease (category 4). In this group of patients a cut-off of 5 fmol $mg^{-1}$ protein was considered positive for ER analysis.

### *Immunohistochemistry*

The tumour tissue was stained with a monoclonal IgM kappa antibody, RTJ1, raised to a synthetic peptide from the cytoplasmic domain of the human c-erbB-3 protein (Rajkumar et al., 1993). A standard avidin–biotin immunochemical technique was used. Sections (3 $\mu$M) were cut from formalin-fixed, paraffin-embedded tumour samples, dewaxed in xylene and taken to alcohol. Endogenous peroxidase activity was blocked with hydrogen peroxide in methanol and non-specific binding sites were blocked with swine serum. Sections were incubated with the RTJ1 antibody at a 1:10 dilution. This dilution was shown to give optimal staining in pilot experiments. Binding of the primary antibody was demonstrated by a standard avidin–biotin complex techique; biotinylated goat anti-mouse immunoglobulin followed by preformed soluble complexes of avidin and biotinylated horseradish peroxidase (Dako). Diaminobenzidine was used as the chromogen with copper sulphate enhancement and haematoxylin was used as the counterstain. Sections were also processed in the absence of RTJ1 antibody to act as negative controls and tumours of known c-erbB-3 immunoreactivity were stained as positive controls on each run.

Staining was assessed according to the degree of cytoplasmic staining on a four-point scale: 0, negative; 1, weakly positive; 2, moderately positive; 3, strongly positive.

Owing to heterogeneous immunoreactivity within most sections, the whole tumour was systematically assessed by grading fields every 0.2 cm within the section. If the tumour area within the section was small or diffuse throughout the stroma then the whole slide was scanned and graded. The overall intensity for each tumour was taken to be that shown by the majority of fields. Blind reassessment of a random 15% of the sections in each series ensured consistency in assessment.

Adjacent normal breast epithelial tissue was also assessed. Immunoreactivity in normal tissue was found to be heterogeneous, and, if present, of weak or moderate intensity. For the purpose of this study only those tumours exhibiting strong positivity were considered to overexpress c-erbB-3.

### *Statistical analysis*

Relationships between variables were sought using chi-squared analyses. Survival data were examined by the life-table method (Mantel–Cox). All statistical analyses were performed using SPSSX software.

### Results

A variable degree of immunoreactivity was observed in breast tumours. Within the carcinomas staining was found to be heterogeneous and predominantly cytoplasmic with membrane staining seen in less than 1% of cases (Figure 1). The majority of tumours in both series exhibited moderate immunoreactivity but a substantial proportion in both the primary and advanced series exhibited strong positivity (Figure 2). The cytoplasmic appearance of the stain varied from finely granular to diffuse.

### *Primary operable breast cancer*

Seventeen of the 346 sections (5%) in the primary series showed no immunoreactivity with the RTJ1 antibody, 111 (32%) were weakly positive, 167 (48%) showed moderate positivity and 51 (15%) were scored as showing strong positivity.

Associations with other prognostic variables and survival are shown in Table I. No correlation was found between c-erbB-3 overexpression and OS, DFI or regional recurrence, age, menopausal status, ER status, histological grade, lymph node stage, the presence of distant metastases, VI and c-erbB-2 protein expression.

An association was seen between the intensity of c-erbB-3 immunostaining with the RTJ1 antibody and factors indicative of poor prognosis in this series of patients with primary operable breast cancer. A trend was seen between c-erbB-3 immunostaining and tumour type group ($P=0.05$); patients in the poor prognostic type group more often showed moderate or strong staining, whereas tumours of

**Figure 1** An invasive adenocarcinoma of the breast showing positive immunoreactivity for c-erbB-3 of varying intensities of all tumour cells.

**Figure 2** An invasive adenocarcinoma of the breast adjacent to a benign mammary duct. The tumour cells show intense c-erbB-3 reactivity. There is very mild heterogeneous reactivity in the normal luminal epithelial and myoepithelial cells.

**Table I** Associations of c-erbB-3 immunostaining (none, weak; moderate or strong) with other prognostic factors, survival and recurrence in patients with primary operable breast carcinoma

| *Factor* | *Cut off* | *Chi-squared* | P |
|---|---|---|---|
| Age | <30, 31–40, 41–50, 51–60, >60 years | 7.0 | 0.86 |
| Menopausal status | | 7.9 | 0.25 |
| ER status | 10 fmol $mg^{-1}$ protein | 6.9 | 0.07 |
| Histological grade | 1, 2, 3 | 11.0 | 0.28 |
| Lymph node stage | 1, 2, 3 | 12.4 | 0.19 |
| Size | <2, 2.1–5.0, >5.0 cm | 18.4 | <0.01 |
| Local recurrence | | 5.76 | 0.12 |
| Regional recurrence | | 6.5 | 0.37 |
| Distant metastases | | 4.5 | 0.22 |
| C-erbB-2 | Membrane immunoreactivity | 4.0 | 0.26 |
| VI | None, probable, definite | 8.4 | 0.21 |
| Tumour type | 1, 2, 3, 4[a] | 16.9 | 0.05 |
| OS | Overall statistic (3 d.f.) | 0.2 | 0.98 |
| DFI | Overall statistic (3 d.f.) | 4.0 | 0.26 |

[a]See Pereiraet al., 1995.

**Table II** Associations of c-erbB-3 immunostaining with other prognostic factors and with survival in patients with advanced breast cancer

| | | *Stages III and IV* | |
|---|---|---|---|
| *Factor* | *Cut off* | *Chi-squared* | P |
| Age | <40, 41–61, >61 years | 1.4 | 0.49 |
| Menopausal status | | <0.1 | 1.00 |
| ER status | 5 fmol $mg^{-1}$ protein | <0.1 | 0.99 |
| Histological grade | 1, 2, 3 | 1.3 | 0.52 |
| C-erbB-2 | Membranous reactivity positive | <0.1 | 0.92 |
| VI | None, probable or definite | 1.3 | 0.53 |
| Survival | | 0.2 | 0.68 |
| UICC categories | 1, 2, 3 *vs* 4 | <0.1 | 0.79 |

excellent prognosis more commonly showed weak immunoreactivity. In addition an association was seen with tumour size ($P<0.01$); primary breast tumours larger than 2 cm in size showed a tendency to be more frequently moderately or strongly positive for c-erbB-3. These associations with tumour size and type were not found if c-erbB-3 was grouped into two categories as negative or positive (weak, moderate or strong) when *P*-values were 0.68 and 0.37 respectively.

Information on local recurrence of disease was available on 323 patients of whom 77 had local recurrence of breast carcinoma. Although no overall relationship between c-erbB-3 immunostaining and local recurrence of disease was identified when tumours were analysed in the four groups of immunoreactivity ($P=0.12$), a correlation between degree of immunoreactivity and local recurrence was seen when those tumours showing no or only weak immunostaining were compared with those showing moderate and strong reactivity. A total of 56 of the 77 patients with local recurrence of disease (72.7%) had tumours which stained moderately or strongly with the RTJ1 antibody compared with 144 of the 246 (58.5%) with no evidence of locally recurrent carcinoma ($P=0.03$).

### *Advanced breast cancer*

Two of the 145 sections (1%) in the advanced series showed no immunostaining, 32 (22%) were classed as weakly positive, 61 (42%) showed moderate positivity and 50 (35%) were categorised as strongly positive.

No associations were demonstrated between c-erbB-3 overexpression and age, menopausal status, ER status, histological grade, c-).

### Discussion

There has been considerable interest in the amplification or regulation of members of the type I family of tyrosine kinase growth factor receptors in human breast carcinoma. The first member of the family, epidermal growth factor receptor (EGFR), a 170 kDa transmembrane glycoprotein, has been shown to play a role in normal breast development and differentiation. Overexpression of EGFR in human breast carcinoma was associated with a poor prognosis by Sainsbury *et al.* (1987). Relapse-free survival and OS were found to be significantly decreased in EGFR-positive tumours. This association with a poor prognosis has been suggested by many studies. EGFR expression has also been correlated with established prognostic indicators, with an important inverse relationship between EGFR and ER status. This suggests a possible role for EGFR in the treatment of breast cancer as well as a prognostic indicator (Sainsbury *et al.*, 1985, 1987; Nicholson *et al.*, 1990).

The second member of the family, c-erbB-2 is a 185 kDa transmembrane glycoprotein. Comparative molecular and immunohistological studies have demonstrated an association between amplification of the c-*erb*B-2 gene and strong cell membrane immunoreactivity for the protein in some solid human tumours and, in particular, human breast carcinoma. Such amplification of the gene, detected either by molecular investigation or through immunocytochemical demonstration of membrane protein has, in large studies examining large series of breast cancers, been shown to be associated with a poorer prognosis. In 1987, Slamon *et al.* carried out an initial study that demonstrated a significant relationship between c-erbB-2 immunoreactivity and a shorter DFI and OS (Slamon *et al.*, 1987). Further studies have supported this association (Wright *et al.*, 1989; Gullick *et al.*, 1991; Lovekin *et al.*, 1991). An inverse association has also been demonstrated between c-erbB-2 expression and ER status (Slamon *et al.*, 1987).

The c-*erb*B-3 gene was first cloned by Kraus *et al.* in 1989 and subsequently by Plowman *et al.* in 1990. Its protein product is a 180 kDa transmembrane glycoprotein that shows considerable sequence homology to the EGFR and the c-erbB-2 protein, especially in the tyrosine kinase domain.

A study examining c-erbB-3 protein expression in normal human adult and fetal tissues demonstrated that most developing human tissues, except haemopoietic tissues, express c-erbB-3 and expression is not restricted to proliferating cells. Normal adult breast tissue shows a moderately intense staining of luminal epithelial cells of breast acini and a weaker reactivity of basal myoepithelial cells. This normal distribution is distinctive and different from that observed with EGFR and c-erbB-2. Reactivity is predominantly cytoplasmic and no membrane reactivity of normal tissue has been observed in these early studies (Prigent *et al.*, 1992).

Poller *et al.* used a polyclonal antibody raised to the c-erbB-3 protein to examine c-erbB-3 expression in a variety of adenocarcinomas. C-erbB-3 protein expression was detected in a series of 13 out of 14 primary breast carcinomas. Expression of the c-erbB-3 protein was found to be a common event in adenocarcinomas but its role in neoplastic progression remained unclear (Poller *et al.*, 1992).

In a more detailed study of breast carcinoma, Lemoine *et al.* (1992) showed consistently higher levels of the c-erbB-3 oncoprotein in cell lines but a wide range of expression in resected primary human breast tumours. In the breast tumours overexpression was seen in 22% of cases, the predominant pattern being cytoplasmic with membrane immunoreactivity being seen in one case only. Investigation of associations with tumour size, histological grade, stage and survival showed correlation only with lymph node metastatic disease. No relationship with overall prognosis was demonstrated.

In our series, using the IgM monoclonal antibody RTJ1 (Rajkumar *et al.*, 1993), we have examined the largest series of patients presenting with breast carcinoma to date, including 346 patients with primary operable breast cancer and 145 patients with advanced disease. Cytoplasmic reactivity was the predominant pattern seen and less than 1% of tumours showed positive membrane reactivity. As previously demonstrated by Lemoine *et al.*, the degree of expression varied (Lemoine *et al.*, 1992). The majority of tumours expressed moderate immunoreactivity. Fifteen percent and 35% of cases in the primary and advanced series respectively demonstrated strong positive staining which for the purposes of this study was considered overexpression, as reactivity in normal breast epithelial tissue, although heterogeneous, was confined to negative, weak or moderate levels of intensity. Different levels of frequency of overexpression were identified in the two groups of patients in this study but this failed to reach statistical significance.

No significant associations were demonstrated between c-erbB-3 expression and survival in either patients with primary operable breast cancer or those with advanced disease and no correlation with DFI was found in the former group. In particular there was no relationship with lymph node status in this series which is in contrast to the study carried out by Lemoine *et al.* (1992). In the patients with operable disease, however, an association was seen between greater intensities of immunoreactivity with RTJ1 antibody and both increased tumour size and a weaker association with poor prognostic type group. In addition those tumours which showed moderate or strong immunoreactivity with c-erbB-3 antibody appeared to be more likely to develop locally recurrent disease. The associations we report here have not been previously documented. Nevertheless, it seems unlikely that immunohistochemical assessment of c-erbB-3 expression will provide sufficiently powerful prognostic information to be clinically useful; the associations with other prognostic factors we describe here are based on differences in intensity of immunoreactivity rather than presence or absence of staining.

A recent study has demonstrated that the growth factor ligand heregulin binds to the c-erbB-3 receptor (Carraway *et al.*, 1994). The same group have also demonstrated little or no tyrosine kinase activity following stimulation and binding of heregulin with the c-erbB-3 receptor. However, in cells expressing both c-erbB-2 and c-erbB-3 a high-affinity binding site is generated and on stimulation produces unique tyrosine residues (Sliwkowski *et al.*, 1994). This is in contrast to the interaction and complex formation between c-erbB-2 and c-erbB-4, where both receptors have active tyrosine kinase components which are capable of autophosphorylation (Plowman *et al.*, 1993). The potential for type I tyrosine kinase receptors to produce different combinations of heregulin-stimulated heterodimeric complexes could explain some of the varied biological activities that have been demonstrated with this group of receptors (Carraway and Cantley, 1994). We have found no association between overexpression of c-erbB-2 and c-erbB-3 assessed immunohistochemically.

In common with other published series we have demonstrated virtually ubiquitous cytoplasmic expression of c-erbB-3 protein at weak to strong levels. The c-*erb*B-3 gene sequence codes for transmembrane types of protein but membrane localisation of c-erbB-3 protein appears to be a rare phenomenon and is much lower in frequency than EGFR and c-erbB-2 proteins in invasive breast cancer. It is known that EGFR and c-erbB-2 are internalised by endocytosis after ligand binding. The predominant cytoplasmic localisation of c-erbB-3 protein could indicate internalised, non-functional or non-membrane-associated protein. The c-erbB-3 protein may be an orphan receptor (Kraus *et al.*, 1993) but, if the parent of the orphan in terms of signalling were c-erbB-2 or c-erbB-4 as has recently been suggested, then the c-erbB-3 protein may be an important cofactor in the biological effects of type I growth factor receptors.

### References

CARRAWAY KL AND CANTLEY LC. (1994). A new acquaintance for erbB3 and erbB-4: a role for receptor heterodimerisation in growth signalling. *Cell*, **78**, 5–8.

CARRAWAY KLI, SLIWKOWSKI MX, AKITA R, PLATKO JV, GUY PM, NUIJENS A, DIAMONTI AJ, VANDLEN RL, CANTLEY LC AND CERIONE RA. (1994). The erbB3 gene product is a receptor for heregulin. *J. Biol. Chem.*, **269**, 14303–14306.

ELLIS IO, GALEA M, BROUGHTON N, LOCKER A, BLAMEY RW AND ELSTON CW. (1992). Pathological prognostic factors in breast cancer. II. Histological type. Relationship with survival in a large study with long-term follow-up. *Histopathology*, **20**, 479–489.

ELSTON CW AND ELLIS IO. (1991). Pathological prognostic factors in breast cancer. I. The value of histological grade in breast cancer: Experience from a large study with long-term follow-up. *Histopathology*, **19**, 403–410.

GASPARINI G, GULLICK WJ, MALUTA S, PALMA PD, CAFFO O, LEONARDI E, BORACCHI P, POZZA F, LEMOINE NR AND BEVILACQUA P. (1994). c-erbB-3 and c-erbB-2 protein expression in node-negative breast carcinoma – an immunocytochemical study. *Eur. J. Cancer*, **30A**, 16–22.

GRIMAUX M, ROMAIN S, REMVIKOS Y, MARTIN PM AND MAGDELENAT H. (1989). Prognostic value of epidermal growth factor receptor in node-positive breast cancer. *Breast Cancer Res. Treat.*, **14**, 77–90.

GULLICK WJ, LOVE SB, WRIGHT C, BARNES DM, GUSTERSON B, HARRIS AL AND ALTMAN DG. (1991). c-erbB-2 protein overexpression in breast cancer is a risk factor in patients with involved and uninvolved lymph nodes. *Br. J. Cancer*, **63**, 434–438.

HAYBITTLE JL, BLAMEY RW, ELSTON CW, JOHNSON J, DOYLE PJ, CAMPBELL FC, NICHOLSON RI AND GRIFFITHS K. (1982). A prognostic index in primary breast cancer. *Br. J. Cancer*, **45**, 361–366.

KRAUS MH, FEDI P, STARKS V, MURARO R AND AARONSON SA. (1993). Demonstration of ligand-dependent signaling by the erbB-3 tyrosine kinase and its constitutive activation in human breast tumor cells. *Proc. Natl Acad. Sci. USA*, **90**, 2900–2904.

LEMOINE NR, BARNES DM, HOLLYWOOD DP, HUGHES CM, SMITH P, DUBLIN E, PRIGENT SA, GULLICK WJ AND HURST HC. (1992). Expression of the ERBB3 gene product in breast cancer. *Br. J. Cancer*, **66**, 1116–1121.

LOVEKIN C, ELLIS IO, LOCKER A, ROBERTSON JFR, BELL J, NICHOLSON R, GULLICK WJ, ELSTON CW AND BLAMEY RW. (1991). c-erbB-2 oncoprotein expression in primary and advanced breast cancer. *Br. J. Cancer*, **63**, 439–443.

NICHOLSON S, WRIGHT C, SAINSBURY JRC, HALCROW P, KELLY P, ANGUS B, FARNDON JR AND HARRIS AL. (1990). Epidermal growth factor receptor (EGFr) as a marker for poor prognosis in node-negative breast cancer patients: Neu and tamoxifen failure. *J. Steroid Biochem. Mol. Biol.*, **37**, 811–814.

PEREIRA H, PINDER SE, SIBBERING DM, GALEA MH, ELSTON CW, BLAMEY RW, ROBERTSON JFR AND ELLIS IO. (1995). Pathological prognostic factors in breast cancer. IV: Should you be a typer or a grader? A comparative study of two histological prognostic features in operable breast carcinoma. *Histopathology*, **27**, 219–226.

PINDER SE, ELLIS IO, GALEA M, O'ROURKE S, BLAMEY RW AND ELSTON CW. (1994). Pathological prognostic factors in breast cancer. III. Vascular invasion: Relationship with recurrence and survival in a large study with long-term follow-up. *Histopathology*, **24**, 41–47.

PLOWMAN GD, GREEN JM, CULOUSCOU JM, CARLTON GW, ROTHWELL VM AND BUCKLEY S. (1993). Heregulin induces tyrosine phosphorylation of HER4/p180 (erbB4). *Nature*, **366**, 473–475.

POLLER DN, SPENDLOVE I, BAKER C, CHURCH R, ELLIS IO, PLOWMAN GD AND MAYER RJ. (1992). Production and characterization of a polyclonal antibody to the c-erbB-3 protein: Examination of c-erbB-3 protein expression in adenocarcinomas. *J. Pathol.*, **168,** 275–280.

PRIGENT SA, LEMOINE NR, HUGHES CM, PLOWMAN GD, SELDEN C AND GULLICK WJ. (1992). Expression of the c-erbB-3 protein in normal human adult and fetal tissues. *Oncogene*, **7,** 1273–1278.

RAJKUMAR T, GODEN CSR, LEMOINE NR AND GULLICK WJ. (1993). Expression of the C-erbB-3 protein in gastrointestinal tract tumours determined by monoclonal antibody RTJ1. *J. Pathol.*, **170,** 271–278.

SAINSBURY JRC, MALCOLM AJ, APPLETON DR, FARNDON JR AND HARRIS AL. (1985). Presence of epidermal growth factor as an indicator of poor prognosis in patients with breast cancer. *J. Clin. Pathol.*, **38,** 1225–1228.

SAINSBURY JRC, FARNDON JR, NEEDHAM GK, NEEDHAM GK, MALCOLM AJ AND HARRIS AL. (1987). Epidermal-growth-factor receptor status as predictor of early recurrence of and death from breast cancer. *Lancet*, **1,** 1398–1402.

SLAMON DJ, CLARK GM, WONG SG, LEVIN WJ, ULLRICH A AND MCGUIRE WL. (1987). Human breast cancer: Correlation of relapse and survival with amplification of the HER-2/neu oncogene. *Science*, **235,** 177–182.

SLIWKOWSKI MX, SCHAEFER G, AKITA RW, LOFGREN JA, FITZPATRICK VD, NUIJENS A, FENDLY BM, CERIONE RA, VANDLEN RL AND CARRAWAY KL. (1994). Coexpression of erbB2 and erbB3 proteins reconstitutes a high affinity receptor for heregulin. *J. Biol. Chem.*, **269,** 14661–14665.

WALKER RA, GULLICK WJ AND VARLEY JM. (1989). An evaluation of immunoreactivity for c-erbB-2 protein as a marker of poor short-term prognosis in breast cancer. *Br. J. Cancer*, **60,** 426–429.

WRIGHT C, ANGUS B, NICHOLSON S, SAINSBURY J, CAIRNS J, GULLICK WJ, KELLY P, HARRIS AL AND WILSON HORNE CH. (1989). Expression of c-erbB-2 oncoprotein: A prognostic indicator in human breast cancer. *Cancer Res.*, **49,** 2087–2090.